
JOURNAL INFORMATION
==============================
NLM Title Abbreviation: Wirtschaftsdienst
Journal ID: Wirtschaftsdienst
NLM Journal ID: 101086612

Wirtschaftsdienst (Hamburg, Germany : 1949)

ISSN: 0043-6275

ARTICLE INFORMATION
==============================
PMCID: PMC8598268
PMID: 34812206
DOI: 10.1007/s10273-021-3042-y
Article ID: 3042
Article version: 1
Subjects: Zeitgespräch

Die Zukunft der EU-China-Handelspolitik: Herausforderungen angehen und eigene Handelsinteressen selbstbewusst vertreten

Flach Lisandra Flach@ifo.de

Teti Feodora teti@ifo.de

grid.469877.3 0000 0004 0397 0846 ifo-Zentrum für Außenwirtschaft, ifo-Institut - Leibniz-Institut für Wirtschaftsforschung an der Universität München e.V., Poschingerstr. 5, 81679 München, Deutschland
Electronic publication date: 2021 Nov 18
Print publication date: 2021
Volume: 101
Issue: 11
First page: 849
Last page: 853
Copyright: © Der/die Autor:in 2021
License: Open Access: Dieser Artikel wird unter der Creative Commons Namensnennung 4.0 International Lizenz veröffentlicht (https://creativecommons.org/licenses/by/4.0/deed.de).
Open Access wird durch die ZBW — Leibniz-Informationszentrum Wirtschaft gefördert.
License URL: https://creativecommons.org/licenses/by/4.0/

issue-copyright-statement: © The Author(s) 2021

==============================
Prof. Dr. Lisandra Flach ist Leiterin des ifo Zentrums für Außenwirtschaft und Professorin für Volkswirtschaftslehre, insbesondere Ökonomik der Globalisierung, an der Ludwig-Maximilians-Universität München.

Die EU braucht eine klare Strategie für die tiefgreifenden und vielschichtigen Handelsbeziehungen mit China. Die Handelsbeziehungen zwischen Europa und China sind von engen wirtschaftlichen Verflechtungen, aber auch komplexen geopolitischen Fragen geprägt. Für den deutschen Güterhandel nimmt China als Hauptimportland sowie als zweiwichtigstes Zielland für Güterexporte eine herausragende Stellung ein.[1] Auch für die EU als

Abbildung 1
**Rückwärtsverflechtungen der EU, USA und China**

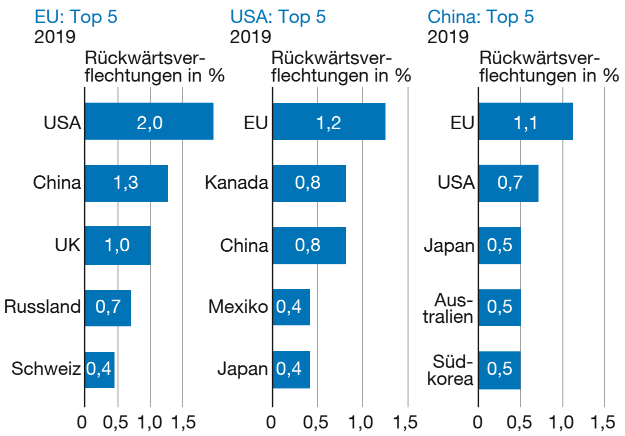

Quelle: Flach et al. (2021a).

Ganzes spielen die Verflechtungen mit China eine wichtige Rolle: Außerhalb der EU sind die USA und China die wichtigsten Zulieferer von Zwischenprodukten (vgl. Abbildung 1). Aber auch für China ist die EU die wichtigste Zuliefererin von Zwischenprodukten, wenn man den Anteil an der Wertschöpfung betrachtet. Vorleistungen aus der EU tragen 1,1 % zur Produktion chinesischer Finalgüter bei (vgl. Abbildung 1).

## Die gegenseitigen Lieferabhängigkeiten zwischen China und der EU

Dieser Zusammenhang verdeutlicht, dass nicht nur die EU von China abhängig ist, sondern auch andersherum – es bestehen also wechselseitige Lieferabhängigkeiten zwischen den beiden Blöcken. Zudem hebt die Analyse globaler Lieferketten die zentrale Rolle der EU für die Weltproduktion und insbesondere für Deutschland hervor. *Erstens* ist Deutschland für viele EU-Mitgliedstaaten einer der wichtigsten Zulieferer. *Zweitens* befinden sich im Gegenzug viele der wichtigsten Zuliefererländer Deutschlands innerhalb der EU. Sie stellen insbesondere Deutschlands mit Abstand wichtigste Bezugsquelle sogenannter abhängiger Güter dar, d. h. Güter, die bei Lieferausfällen nur schwer ersetzbar wären. *Drittens* spielt zwar Deutschland allein betrachtet als Zulieferer für China mit einem Wert von 0,4 % der Zwischenprodukte nur eine untergeordnete Rolle (Flach et al., 2021a), die EU als Ganzes betrachtet ist jedoch der wichtigste Zulieferer für China und die USA.

Um das Risiko von Engpässen in der Lieferkette bewerten zu können, ist es wichtig, die Abhängigkeiten in der Lieferkette auf Produktebene zu kennen. Fast drei Viertel aller deutschen Industriegüter, bei denen Abhängigkeiten bestehen, stammen aus EU-Ländern (Flach et al., 2021a). Aus China kommen im Gegensatz dazu lediglich 3 % der abhängigen Waren. Viele davon sind in der Chemie- oder Elektroindustrie zu finden, aber auch in Branchen wie dem Maschinenbau oder bei Metallprodukten gibt es Beispiele von Gütern, die besonders abhängig von chinesischen Zulieferern sind. Eine stärkere Diversifizierung der Lieferkette ist der beste Weg, um diese Abhängigkeiten zu reduzieren. Wünschenswert und auch umsetzbar wäre dies z. B. bei Arzneimitteln; schwieriger wird es hingegen etwa bei natürlichen Ressourcen, da diese häufig nur in einer begrenzten Zahl an Ländern vorkommen. Insbesondere in solchen Fällen ist die Identifizierung und Überwachung von Risiken in Lieferketten besonders wichtig. Dabei können Unternehmen von staatlicher Seite unterstützt werden, z. B. durch die Förderung von digitalen Technologien im Bereich des Risikomanagements. Auch Handelsabkommen sind ein möglicher Weg, um Unsicherheiten zu reduzieren.

## Kehrt China dem Weltmarkt den Rücken zu?

Die COVID-19-Pandemie und die resultierenden Schwierigkeiten in der globalen Produktion sowie die von Trump initiierten Handelskonflikte haben die Forderungen nach einer Verringerung der Abhängigkeit vom globalen Markt verstärkt und mögliche Schwachstellen des bisherigen exportorientierten Wachstumsmodells offengelegt. Der aktuelle Fünfjahresplan Chinas, der im Frühjahr 2021 vorgestellt wurde, spiegelt diese Erkenntnisse wider und gibt Aufschluss über die Marschrichtung, die die kommunistische Partei einschlagen möchte, um auf diese Entwicklung zu reagieren. Als übergeordnete Ziele definiert die chinesische Regierung nachhaltiges Wirtschaftswachstum, das vor allem durch eine Stärkung der Binnennachfrage erreicht werden soll, sowie eine von Innovation angetriebene Industrie und Landwirtschaft (Botschaft der

VR China, 2021). China plant also, die bisherige starke Abhängigkeit von der Weltkonjunktur und des Welthandels, insbesondere von der Nachfrage aus den USA und Europa nach chinesischen Waren sowie essenziellen Inputs, zu reduzieren.

Das bedeutet aber keinesfalls, dass China zurück in die Autarkie möchte. Stattdessen sollen Risiken verringert werden, indem die Handelsbeziehungen mit anderen Partnern gefördert werden. Vor allem die Kooperation mit den asiatischen Nachbarn soll gestärkt werden. Die Unterzeichnung des RCEP-Abkommens im November 2020 stellt einen wichtigen Schritt in diese Richtung dar. Neben China sind die zehn ASEAN-Staaten (Brunei, Indonesien, Kambodscha, Laos, Malaysia, Myanmar, die Philippinen, Singapur, Thailand und Vietnam), Japan, Südkorea sowie Australien und Neuseeland Teil des Handelsabkommens. Das Abkommen umfasst somit 15 Mitgliedsstaaten, auf die 28 % der Weltwirtschaftsleistung, 28 % des Welthandels und 29 % der Weltbevölkerung entfallen (Hildenbrand und Teti, 2021).

Insbesondere für den Handel Chinas mit Japan bzw. Südkorea ist ein starker Anstieg zu erwarten, da zusätzlich zur Reduktion von nicht tarifären Handelsbarrieren die Zölle um im Durchschnitt 8 Prozentpunkte sinken sollen. Außerdem wird die Harmonisierung der Ursprungsregeln, welche bisher mit einem hohen bürokratischen Aufwand für Exporteure verbunden sind, zu robusteren Lieferketten innerhalb des RCEP-Raums führen (Flach et al., 2021b). Die Ausgestaltung des RCEP-Abkommens zeigt, dass die wirtschaftliche Integration des asiatischen Raums noch stärker vorangetrieben werden soll – mit einem besonderen Augenmerk auf den Ausbau regionaler Lieferketten.

Aber auch mit anderen Handelspartnern Chinas wie den beiden EFTA-Mitgliedern Norwegen und Island oder auch den Golfstaaten (Saudi-Arabien, Vereinigte Arabische Emirate, Kuwait, Oman, Katar und Bahrain) sowie kleineren Staaten wie Sri Lanka, Israel oder Moldawien laufen aktuell Verhandlungen zu Handelsabkommen. China versucht also, Risiken zu diversifizieren und seine Handelsbeziehungen mit neuen Partnern auszubauen. Allerdings konzentriert sich die aktuelle handelspolitische Agenda vor allem auf Staaten, die im Vergleich zu China kleinere Volkswirtschaften sind, welche in besonderem Maße vom Zugang zum chinesischen Markt profitieren und deshalb in der schwächeren Verhandlungsposition sind.

## Handelspolitik zwischen China und der EU

Die handelspolitischen Beziehungen zur EU gehen indes nicht über die multilateralen Vereinbarungen, denen China mit dem Beitritt zur WTO im Jahr 2001 zugestimmt hat, hinaus.[2] Tabelle 1 zeigt die durchschnittlichen sektoralen Zölle, die für Exporte nach China bzw. in die EU anfallen. China erhebt im Vergleich zur EU deutlich höhere Zölle: Für das Jahr 2017 betrug der Zoll über alle Produkte hinweg im Durchschnitt 11,6 %, im Gegensatz zu europäischen Zöllen von nur durchschnittlich 5,3 %. Vor allem bei Agrarprodukten, aber auch bei Textilien (Leder, Schuhe, Spinnstoffe und Bekleidung) sowie Steinen, Glas und Beförderungsmitteln schützt die chinesische Regierung ihre heimische Produktion durch hohe Zölle. Während in China die Zölle in allen Sektoren hoch sind, kann man bei den europäischen Zöllen auf chinesische Produkte starke Schwankungen über die Sektoren hinweg beobachten; vor allem im Agrar- und Textilsektor fallen auch beim Export in die EU hohe Zölle an.

Tabelle 1
### Durchschnittliche Zölle Chinas und der EU, 2017
in %

| Sektor | China | EU |
|---|---|---|
| Lebende Tiere | 13,0 | 19,1 |
| Waren pflanzlichen Ursprungs | 15,1 | 11,1 |
| Fette und Öle | 13,8 | 9,0 |
| Lebensmittel, Getränke und Tabak | 18,6 | 17,9 |
| Mineralische Stoffe | 3,7 | 0,3 |
| Chemische Industrie | 16,8 | 4,7 |
| Kunststoffe | 9,2 | 4,7 |
| Leder | 13,8 | 2,1 |
| Holz | 4,8 | 2,5 |
| Papier und Pappe | 5,7 | 0,2 |
| Spinnstoffe und Bekleidung | 11,5 | 7,9 |
| Schuhe | 18,3 | 7,3 |
| Steine und Glas | 13,7 | 3,4 |
| Schmuck | 9,8 | 0,6 |
| Unedle Metalle | 8,2 | 2,0 |
| Mechanische Geräte und elektrotechnische Waren | 8,6 | 1,9 |
| Beförderungsmittel | 11,2 | 4,0 |
| Optische Instrumente | 10,7 | 2,4 |
| Waffen und Munition | 13,0 | 2,5 |
| Verschiedene Waren | 12,9 | 2,5 |
| Kunstgegenstände und Antiquitäten | 8,8 | 0 |
| | | |
| Mittelwert | 11,6 | 5,3 |

Quelle: Teti (2020).

2 Da auch die USA kein Handelsabkommen mit China haben, gelten – zumindest in normalen Zeiten – dieselben Bedingungen wie für die EU.

Das bedeutet aber keinesfalls, dass die EU ein Hort des Freihandels ist: Insbesondere nicht tarifäre Handelsbarrieren erschweren den Handel. So machen beispielsweise Subventionen und exportbezogene Maßnahmen wie etwa im EU-Agrarsektor knapp über 60 % aller weltweit verhängten protektionistischen Maßnahmen aus (Evenett und Fritz, 2020). Im Agrarsektor gibt es in der EU enorme Subventionen für Landwirte und mengenmäßige Einfuhrbeschränkungen für viele Produkte, die mit hohen Kosten für den EU-Haushalt verbunden sind und zu Wettbewerbsverzerrungen führen. Auch sind Antidumpingzölle ein probates Mittel, um sich gegen die chinesische Konkurrenz zu schützen. Im Jahr 2019 waren 51 % (61 Fälle) aller Antidumpingmaßnahmen der EU gegen China gerichtet (Garcia-Herrero et al., 2020).

Ein tiefgreifendes Handelsabkommen zwischen den beiden Handelspartnern könnte also die Handelskosten für beide Seiten deutlich reduzieren. Zwar macht die Kommission in der neuen EU-Handelsstrategie deutlich, dass der „Aufbau einer fairen und regelbasierten Wirtschaftsbeziehung mit China von vorrangiger Bedeutung ist“ (European Commission, 2021). Ein bilaterales Abkommen ist jedoch ein Wunsch in weiter Ferne – zu groß sind die Differenzen zwischen den Handelspartnern hinsichtlich fundamentaler Aspekte wie der starken Marktzugangsbeschränkungen Chinas, dem unzureichenden Schutz von geistigem Eigentum oder auch der Verletzung von Menschenrechten. Die Schwierigkeiten bei der Ratifizierung des Investitionsabkommens zwischen der EU und China, das weniger tiefgreifend ist als ein Handelsabkommen und bei dem man deshalb leichter zu einer Einigung kommen sollte, verdeutlichen die fundamentalen Unterschiede zwischen den beiden Wirtschaftsmächten.

## Multilateralismus als goldener Weg

Ähnlicher Kritik muss sich China auch auf internationaler Bühne stellen. Bei der diesjährigen Überprüfung der Handelspraktiken Chinas durch die WTO machten einige Partner ihrem Unmut über die unfairen Handelspraktiken im Reich der Mitte Luft. Unter anderem Australien, Japan, Kanada, die EU und die USA prangerten vor allem die staatliche Einmischung in Form von Subventionen oder chinesischen Staatsbetrieben (State-Owned Enterprises – SOEs) an, außerdem die besonders im vergangenen Jahr zunehmende Zahl undurchsichtiger und nicht-WTO-konformer protektionistischer Maßnahmen wie Importverbote unter dem Deckmantel des Verstoßes gegen sanitäre und phytosanitäre Standards. Auch der mangelnde Schutz geistigen Eigentums sowie Zwangsarbeit wurden kritisiert (WTO, 2021). Chinas Handelspraktiken sind zum derzeitigen Zeitpunkt zum Teil unvereinbar mit denen der westlichen Marktwirtschaften. Der ideale Weg, um mit China umzugehen, ist jedoch die multilaterale Zusammenarbeit. Dabei sollte die EU insbesondere gemeinsam mit den USA eine WTO-Reform anstoßen, bei der auch die unfairen Handelspraktiken Chinas adressiert werden.

Seit der Gründung der WTO und dem Abschluss der Uruguay-Runde im Jahr 1996, konnte die WTO nur wenige erwähnenswerte Erfolge verbuchen – es kam zu einem Stillstand des Multilateralismus. Stattdessen besannen sich die Länder auf bilaterale Verhandlungen, und die Zahl bilateraler präferentieller Handelsabkommen schoss in die Höhe. Gleichzeitig ist aber auch ein Anstieg der Zahl der protektionistischen Maßnahmen weltweit zu beobachten. Daten vom Global Trade Alert (Evenett und Fritz, 2020) zeigen, dass seit 2009 die Zahl der protektionistischen Maßnahmen die der liberalisierenden Maßnahmen übersteigt, wobei 2018 ein starker Anstieg zu verzeichnen war. Im Jahr 2019 übertrafen die neu hinzugekommenen protektionistischen Maßnahmen die liberalisierenden Maßnahmen um das 3,7-Fache.

Da sie nicht aktiv an früheren GATT/WTO-Verhandlungsrunden teilgenommen hatten, blieben die Einfuhrzölle in Ländern wie China hoch (Goldberg und Pavcnik, 2016). Wie oben bereits beschrieben liegt der durchschnittliche Zollsatz, der bei der WTO als angewandter Meistbegünstigungszollsatz hinterlegt ist, in China bei 11,6 %, während die Zölle in den Industrieländern viel niedriger sind: 4,4 % in den USA, 4,8 % in Japan und 5,3 % in der EU (Teti, 2020). Das große Zollgefälle macht künftige Zollsenkungen sehr viel schwieriger, da die Industrieländer bei Verhandlungen mit Schwellenländern über Zollsenkungen kaum weitere Zollsenkungen anbieten können. Es ist jedoch wichtig zu erwähnen, dass auch die EU ihre politischen Instrumente, die weit über Zölle hinausgehen, wie z. B. Subventionen überdenken muss.

## Die Rolle der nächsten Bundesregierung

Der Umgang mit China und die resultierenden geopolitischen Implikationen sollten für die künftige Handelspolitik der EU eine zentrale Rolle einnehmen. Zwar werden die meisten handelspolitischen Themen auf EU-Ebene entschieden, aber Deutschland kann und sollte eine aktivere Rolle auf der internationalen Bühne einnehmen. Deutschland macht allein knapp 25 % des BIP der EU aus und der Brexit hat die Stimme Deutschlands innerhalb der EU noch wichtiger gemacht. Deswegen kommt der neuen deutschen Bundesregierung eine wichtige Rolle zu.

In diesem Zusammenhang untersuchen wir die Wahlprogramme für die Bundestagswahl 2021 in Bezug auf die Positionen der Parteien zu China. Während die Parteien in mehreren handelspolitischen Aspekten wie z. B. der Ratifizierung von Freihandelsabkommen stark divergieren, ähneln sich viele Aussagen zu den chinesischen Handelspraktiken. So schreiben die Freien Demokraten, dass wirtschaftliche Zusammenarbeit vertieft, aber Menschenrechtsverletzungen und Marktverzerrungen schonungslos adressiert werden sollten. Beim EU-China-Investitionsabkommen gäbe es zu viele offene Fragen, sodass das Abkommen derzeit nicht ratifiziert werden könne (FDP, 2021). Die Grünen sprechen sich für eine Ablehnung des EU-China-Investitionsabkommens aus, vor allem wegen ungerechter Wettbewerbsbedingungen und der Verletzung von Menschenrechten (BÜNDNIS 90/DIE GRÜNEN, 2021). Die SPD bleibt hingegen relativ vage in ihrem Wahlprogramm und schreibt, dass Europa „den Dialog mit China über Kooperation und Wettbewerb geschlossen, konstruktiv und kritisch führen“ muss (SPD, 2021). Die CDU betont, dass es wichtig sei, das geistige Eigentum, Hochtechnologien und Daten besser zu schützen, um gefährliche Abhängigkeiten zu vermeiden (CDU/CSU, 2021). Auch wenn die Parteien in ihrer Herangehensweise divergieren, haben die Wahlprogramme in Bezug auf China eine sehr ähnliche Sichtweise.

## Die Kosten einer globalen Entkopplung

Die Kosten eines wirtschaftlichen Rückzugs aus der Globalisierung wären für die deutsche Wirtschaft enorm. In einer ifo-Studie im Auftrag der Konrad-Adenauer-Stiftung zeigen wir, dass sowohl der Rückzug aus der Globalisierung (Reshoring) als auch die Rückverlagerung der Produktion nach Deutschland oder in benachbarte Länder, wie beispielsweise in andere EU-Mitgliedstaaten (Nearshoring), starke negative Auswirkungen auf die deutsche Wirtschaft hätten: Bei Reshoring würde das deutsche reale Bruttoinlandsprodukt (BIP) um 9,7 % zurückgehen, bei Nearshoring um 4,2 % (Flach et al., 2021a). Selbst China würde nicht durch das deutsche Reshoring und auch nicht durch das Nearshoring profitieren, denn Deutschland und die EU27 zählen zu den wichtigsten Handelspartnern Chinas. In absoluten Zahlen würden beim Reshoring die deutschen Importe aus China sogar am stärksten zurückgehen.

Zwar legen wir in unserer Studie dar, dass für die deutsche Wirtschaft ein hohes geopolitisches und wirtschaftspolitisches Risiko insbesondere durch China besteht. Allerdings zeigt eine Analyse der Produktabhängigkeiten von China, dass die deutsche Lieferkette ein hohes Maß an Diversifizierung aufweist, sodass die Risiken unter unseren Annahmen eine generelle Entkopplung von globalen Lieferketten nicht rechtfertigen.

Diese erheblichen negativen Auswirkungen machen noch deutlicher, wie dringend notwendig es für die EU ist, einen konstruktiven, aber kritischen Dialog über die Zusammenarbeit und den Wettbewerb mit China zu führen.


Dr. Feodora Teti ist stellvertretende Leiterin des ifo Zentrums für Außenwirtschaft.


Literatur

Aichele R Flach L Braml M Status quo und Zukunft globaler Lieferketten ifo Schnelldienst 2020 73 5 16 22
Botschaft der VR China (2021), Newsletter der Chinesischen Botschaft in Deutschland — Sonderausgabe 14. Fünfjahresplan“ http://de.china-embassy.org/det/zt/Newsletter/P020210416846467419497.pdf (3. November 2021).
BÜNDNIS 90/DIE GRÜNEN (2021), Deutschland. Alles ist drin. Bundestagswahlprogramm 2021, https://cms.gruene.de/uploads/documents/Wahlprogramm-DIE-GRUENEN-Bundestagswahl-2021_barrierefrei.pdf (3. November 2021).
CDU/CSU (2021), Das Programm für Stabilität und Erneuerung. Gemeinsam für ein modernes Deutschland, https://online.fliphtml5.com/kxyi/eyjg/#p=9 (3. November 2021).
European Commission (2021), Trade Policy Review — An Open, Sustainable and Assertive Trade Policy, Communication to the European Parliament, European Commission Press Release, 18. Februar.
Evenett, S. und J. Fritz (2020), The Global Trade Alert Database Handbook, Manuscript, 14. Juli.
FDP (2021), Nie gab es mehr zu tun. Wahlprogramm der Freien Demokraten, https://www.fdp.de/sites/default/files/2021-08/FDP_BTW2021_Wahlprogramm_1.pdf (3. November 2021).
Flach, L., J. Gröschl, M. Steininger, F. Teti und A. Baur (2021a), Internationale Wertschöpfungsketten — Reformbedarf und Möglichkeiten, Studie im Auftrag der Konrad-Adenauer-Stiftung e. V.
Flach L Hildenbrand H Teti F The Regional Comprehensive Economic Partnership Agreement and Its Expected Effects on World Trade Intereconomics 2021 56 2 2021 10.1007/s10272-021-0960-2
Garcia-Herrero, A., G. Wolff, J. Xu, N. Poitier, G. Felbermayr, R. Langhammer, W-.H Liu und A. Sandkamp (2020), EU-China Trade and Investment Relations in Challenging Times, European Parliament, Mai.
Goldberg, P. und N. Pavcnik (2016), The Effects of Trade Policy, Chap. 3, in Handbook of Commercial Policy, Vol. 1A, 161–206, Elsevier B. V.
Hildenbrand, H. und F. Teti (2021), RCEP, die größte Freihandelszone der Welt — Was beinhaltet der Mega-Deal, und mit welchen Auswirkungen ist zu rechnen, ifo Schnelldienst, 74(2).
SPD (2021), Aus Respekt vor deiner Zukunft. Das Zukunftsprogramm der SPD, https://www.spd.de/fileadmin/Dokumente/Beschluesse/Programm/SPD-Zukunftsprogramm.pdf (3. November 2021).
Teti, F. (2020), 30 Years of Trade Policy: Evidence from 5,7 Billion Tariffs, ifo Working Paper, Nr. 334.
WTO (2021) Trade Policy Review China: Concluding Remarks by the Chairperson, https://www.wto.org/english/tratop_e/tpr_e/tp515_crc_e.htm (3. November 2021).
